# Facilitating Peer Interaction Regulation in Online Settings: The Role of Social Presence, Social Space and Sociability

**DOI:** 10.3389/fpsyg.2022.793798

**Published:** 2022-04-25

**Authors:** Emmy Vrieling-Teunter, Maartje Henderikx, Rob Nadolski, Karel Kreijns

**Affiliations:** Faculty of Educational Sciences, Open Universiteit, Heerlen, Netherlands

**Keywords:** self-regulation, online learning, peer interaction, social presence, social space, sociability, higher education

## Abstract

A plethora of studies stress students’ self-regulated learning (SRL) skills to be conditional for successful learning in school and beyond. In general, self-regulated learners are actively engaged in constructing their own understanding also including the regulation of contextual features in the environment. Within the contextual features, the regulation of peer interaction is necessary, because college courses increasingly require peer learning. This goes along with the increasing interest for online learning settings, due in no small part to the recent COVID-19 pandemic. In the present study we explore how social presence (i.e., the degree to which the other person is perceived as physical “real”), social space (i.e., trust building between peers) and sociability (i.e., the degree to which the virtual learning environment supports social presence and social space) are essential elements in the regulation of online peer interaction. To shed light in this matter, higher education students were qualitatively followed for 1 year in an online academic writing course by using retrospective interviews (*n* = 7) and reflective questions (*n* = 62). Additionally, for social presence, students’ perceptions were quantitatively measured with a validated questionnaire (*n* = 41). The results show that the planning phase is the most important phase for supporting students’ social presence because that is where the regulation of peer interaction becomes important. The sociability has an important role here as well becoming less prominent further on in the self-regulation process. In the SRL follow-up phases, students look for other ways to increase their social presence and social space in order to shape the regulation of peer interaction from a position of trust. In the evaluation phase, students are aware of the importance of social presence but less of social space for the regulation of peer interaction. We conclude with some design principles to facilitate students’ regulation of peer interaction in online settings.

## Introduction

### Self-Regulated Learning and Peer Interaction

From a social constructivist point of view, the benefits for students to be actively engaged in constructing their own understanding is generally acknowledged (Power, 2016). One of the shared assumptions of social constructivist learning theories is the significance of self-regulated learning (SRL) as the key component for successful learning in school and beyond (Boekaerts, 1999; Zimmerman, 2001). In general, SRL is defined as “an active, constructive process whereby students set goals for their learning and then attempt to monitor, regulate, and control their cognitions, motivation, and behavior, guided and constrained by their goals and the contextual features in the environment” (Pintrich, 2000, p. 453). Much empirical evidence showed that SRL is of great value for students’ academic success (Hmelo-Silver et al., 2007). Consequently, SRL is gaining attention and teachers in various educational contexts strive to equip students with SRL skills to become adaptive learners and employees (Eccles and Wigfield, 2002). In line with several models of SRL, but particularly in Pintrich’s (2000, 2004) model, students must deal with four phases and four areas for regulation. The four phases include (1) forethought, planning and activation, (2) monitoring, (3) control, and (4) reaction and reflection. The four areas for regulation include (a) cognition (e.g., knowledge activation, knowledge of strategies), (b) motivation and affect (e.g., achievement goals, achievement attributions, self-efficacy), (c) behavior (e.g., time, effort), and (d) context (resources, social context).

When taking a closer look at the areas of SRL, the cognition, motivation and behavior of students cannot be comprehended unless social and cultural context, such as support from teachers and feedback from peers, are taken into consideration (Järvelä et al., 2008). Contextual regulation involves the learner’s own behavior, but it also involves contextual control because it necessarily pertains to the procurement of help from others in the environment which can be regarded as social interaction (Pintrich, 2004). Social interaction regulation involves efforts to control and regulate the context the student encounters in the classroom (Pintrich, 2004). A prevalent form of social interaction regulation concerns the way students interact with peers and construct knowledge in collaboration with peers, that resembles the peer management strategy as described by Dignath et al. (2008). This peer management or peer interaction regulation pertains to the procurement of help from peers in the environment for example through the use of peer feedback (Eggers et al., 2021). Providing peer feedback is a reciprocal process whereby students produce feedback on the work of peers and receive feedback from peers on their own work (Topping, 1998). Liu and Carless (2006) found that the process of peer feedback increased students’ active learning, self-management and judgement, capacity for self-assessment, and subject knowledge. It also resulted into faster feedback and increased peer interaction. The concepts of peer interaction regulation or peer management can also be compared to the concept of co-regulation (Järvelä et al., 2008). However, co-regulation requires peers to be aware of each other’s goals and progress and to take them into account in the joint task which emphasizes support for each other’s SRL, while peer interaction regulation and peer management primarily highlight working with peers toward individual goals.

### Regulation of Online Peer Interaction

The increased opportunities in the use of the Internet over the past decade have caused traditional face-to-face higher education to create more opportunities for students to engage in online learning (Greenland and Moore, 2014). This goes along with the increasing interest for the social wellbeing of students in online learning settings, largely due to the recent COVID-19 pandemic (Paterson and Prideaux, 2020; Vrieling-Teunter et al., 2021). Contrary to traditional learning where peer interaction occurs face-to-face in a classroom, online learning involves the use of asynchronous and synchronous peer interaction within a virtual learning environment (VLE; Ku and Chang, 2011). Online learning has several advantages over traditional settings because it provides flexibility and accessibility for students, increases access to learning resources, and provides greater opportunities for peer interaction (Broadbent and Poon, 2015). Also, besides synchronous education where students interact and learn at the same time and place, the VLE provides opportunities for asynchronous learning in which space and time are not barriers for peer interaction (Ku and Chang, 2011). In line with the findings in face-to-face learning environments, the review study of Broadbent and Poon (2015) showed that peer interaction regulation in online higher education is positively related to academic achievement.

Despite these advantages, study success in VLEs heavily depends on students’ abilities to self-regulate their learning (Eggers et al., 2021). Students in online courses are responsible for their own learning as they decide when, where, and how long to access the learning materials (McMahon and Oliver, 2001). Therefore, SRL is especially important when taking online courses (Wijekumar et al., 2006) and more attention to understanding SRL in online higher education is crucial (Eggers et al., 2021). Earlier research in the area of SRL and peer interaction, had a strong cognitive perspective (e.g., the use of peer feedback for cognitive benefits). Since the regulation of peer interaction is part of a collaborative process between at least two peers within a larger group of learners, the inclusion of a social perspective would be relevant for the learner benefits and conditions for learning (van Popta et al., 2017). Broadbent and Poon (2015) even argue that peer interaction regulation should be prioritized while examining SRL in online higher education because of its strong effect on study success.

### Social Space, Social Presence, and Sociability

When feedback is provided by peers, perceived relatedness is of importance (van Popta et al., 2017). Relatedness is associated with the construct of social space which can be defined as “the network of interpersonal relationships embedded in group structures of norms and values, rules and roles, and beliefs and ideals” (Kreijns et al., 2021, p. 2). In fact, relatedness is one of the facets of social space being part of the framework of Kreijns et al. (2021) that also encompasses social presence and sociability. When the social space is sound, it will be characterized by attributes like a sense of community, positive group climate, mutual trust, social identity, and group cohesion. In essence, these attributes are reflected in relatedness as well. It is to be noted that a sound social space can be established in face-to-face and online learning settings. Yet, social interaction regulation is needed for peer interaction as well as for developing a sound social space (Kreijns et al., 2021).

In an online learning setting, productive social interaction regulation (effective peer interaction) is much more difficult to achieve because all communication is taking place through the various communication media made available by the electronic learning environment in use (Bromme et al., 2005; Kreijns, 2020). If for instance students do not know each other before they are involved in a course that incorporates peer interaction, the establishment of good interpersonal relationships and a sound social space for creating a safe environment in which peer feedback can be given, takes much longer than in face-to-face learning settings (Wang et al., 2013). Despite these difficulties, peer feedback is important for students to stay connected in online courses (van Popta et al., 2017) and to enhance community building (Corgan et al., 2004). Through peer feedback, students interact in their peers’ learning and thus achieve greater understanding and appreciation for their peers’ experiences and perspectives (van Popta et al., 2017).

Besides social space, a critical construct for social interaction regulation is social presence, defined as “the psychological phenomenon in which, to a certain extent, the other persons are perceived as physical ‘real’ persons in technology-mediated communication enabled by Computer Mediated Communication (CMC) tools and electronic platforms” (Kreijns et al., 2021, p. 2). Many social presence researchers purport that if there is no social presence it will be impossible to have social interaction (e.g., Tu and McIsaac, 2002). It is noted that the perception of social presence depends on a contingency of factors that may have situational and temporal influence on this perception. For instance, when students do not know each other, the influence of video communication media on perceptions of social presence may be huge in the beginning but that influence may diminish when in due course students get to know each other well; that is, when they have created individuated impressions of each other (Walther, 1992, 1996). In that case, other factors may become more important such as the topic of the discussions, the communication style, and the shared history (Tu, 2000a,b; Kreijns, 2020).

Given the relationship between social presence, social interaction and social space (Kreijns, 2020), it is important that the CMC tools and electronic platforms that together form the VLE facilitate social presence perceptions and the emergence of a social space to secure social interaction regulation (effective peer interaction). This is referred to as the sociability of the VLE and is defined as “the capacity of CMC tools and electronic platforms to allow for the expression of social presence and the experience of it as well as for the emergence of a social space” (Kreijns et al., 2021, p. 2).

**FIGURE 1:**
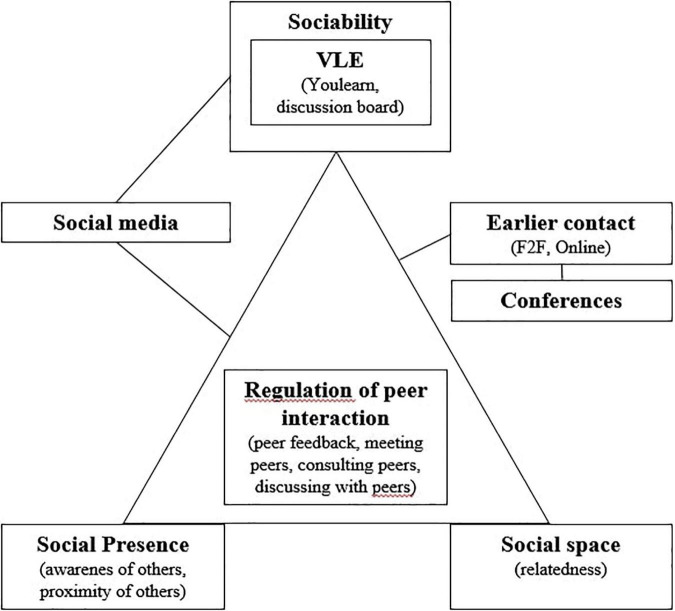
The social space, social presence, and sociability triangle (adapted from Kreijns et al., 2021).

The three constructs, social space, social presence, and sociability, do not work in isolation: together, they influence how the regulation of social interaction (i.e., peer interaction) is established and maintained. Figure 1 visually positions the constructs as a triangle where the sides represent the interrelations and the vertices the constructs social space, social presence, and sociability; the regulation of social interaction (i.e., peer interaction, self-regulation) is positioned in the center of the triangle to reflect that it is affected by those three constructs. In this article, the focus is on the role of social space, social presence and sociability in the regulation of students’ peer interaction.

### Facilitating Regulation of Online Peer Interaction

In the context of the COVID-19 pandemic, designing VLEs has become increasingly important (Paterson and Prideaux, 2020). Despite the fact that attention to SRL is essential for students, SRL research in online environments is scarce and offers few design guidelines (Eggers et al., 2021). To realize high-quality online educational designs, it is therefore important to facilitate instructors in monitoring and evaluating the way in which students effectively organize SRL in online environments (Paterson and Prideaux, 2020). Although the regulation of peer interaction is an important contributor to student success in online higher education, attention to peer interaction in SRL research has been limited (Broadbent and Poon, 2015). Therefore, it is important to gain insight into the optimal conditions for students’ regulation of peer interaction in online higher education. It is necessary to provide an explication of the “teacher as designer’ to facilitate students” regulation of peer interaction. In this search for guidelines, it is important to take the constructs of social space, social presence and sociability into account. It is also important to find out how students develop through the four SRL stages (see section “Self-Regulated Learning and Peer Interaction”) as this may facilitate a gradual development from teacher control to student control over learning processes, also known as scaffolding (Vrieling-Teunter et al., 2018). When undertaking peer interaction regulation, phase 1 involves the planning of peer interaction, phase 2 the monitoring of peer interaction, phase 3 the controlling of peer interaction, and phase 4 the evaluation of peer interaction.

### Research Questions

In order to develop interventions for teachers to optimize social space, social presence and sociability in VLEs for the benefit of online peer interaction regulation, we need to identify the student perceptions and experiences of these concepts. This brings us to the following overall research question: How are social space, social presence, and sociability supportive for peer interaction regulation in VLEs? From the scaffolding perspective, it is also important to examine how the three concepts of social space, social presence, and sociability are experienced by students during peer interaction regulation in the four SRL phases. This results into the following four research questions:

1. How are social space, social presence, and sociability supportive for peer interaction regulation in VLEs in SRL phase 1 (planning of peer interaction)?
2. How are social space, social presence, and sociability supportive for peer interaction regulation in VLEs in SRL phase 2 (monitoring of peer interaction)?
3. How are social space, social presence, and sociability supportive for peer interaction regulation in VLEs in SRL phase 3 (controlling of peer interaction)?
4. How are social space, social presence, and sociability supportive for peer interaction regulation in VLEs in SRL phase 4 (evaluation of peer interaction)?

## Materials and Methods

### Participants and Context

Participants were Belgium and Dutch students of the Open Universiteit of the Netherlands–a distance education institution–who were following the “Trends in education and educational sciences” course (Trends course). This course is a self-paced academic writing course, meaning that students can start this course at any moment during the academic year. Students can therefore study at their own pace within their respective registration period. In addition, students have the freedom to choose their own conferences to visit physically, as long as these conferences adhere to certain scientific level criteria (during the COVID-19 pandemic physical attendance of conferences was not possible, therefore these students attended conferences online). The course consists of three sections during which students have to write three scientific conference reviews. Such open-ended learning tasks require from students to adequately self-regulate their learning (Bennett et al., 2018). The teacher support in the course progressively decreases. During section one and two, the feedback is given by the teacher, based on a rubric, as a combination of scores and comments. During section three, students provide peer feedback in pairs based on the same criteria used in the earlier course sections but limited to comments instead of grades. Finally, similar to the first two assignments, the work is assessed by the teacher. All the course work and interaction take place in a VLE called “YouLearn” which is developed by the Open Universiteit Netherlands. YouLearn offers all the basic functionalities that VLEs generally provide, such as a course structure, a discussion board, the possibility to ask questions, the ability to submit assignments and receive teacher feedback and grades. A total of 41 students (32 women, 9 men) who were enrolled in the course in July and August 2021, completed the questionnaire about social presence (see sections “Materials and Procedure”). Since April 2020, a total of 62 students (46 women, 16 men) who completed the peer feedback assignment, answered three supplementary reflective questions (see sections “Materials and Procedure”). In addition, seven students (four women, three men) participated in semi-structured in-depth interviews (see sections “Materials and Procedure”).

### Materials

Social presence was measured using the scale developed and validated by Kreijns et al. (2020). This scale measures two dimensions of social presence: (1) awareness of others (15 items, e.g., “It feels like as if I deal with ‘real’ persons and not with abstract anonymous persons”) and (2) proximity of others (12 items, e.g., “It feels as if all my fellow students and I are in the same room”). The items were translated to Dutch by two independent researchers following a back translation procedure for validation purposes (Tyupa, 2011). Items were scored on a five-point Likert scale, 1 = “totally disagree” and 5 = “totally agree.”

To find out if or to what extent social presence, social space and sociability were supportive for peer interaction in the four phases of SRL, students were asked to answer three reflective questions as part of the peer feedback assignment (Honold, 2006). An example of a reflective question is: “What was the most valuable learning outcome of the peer feedback assignment for you?” In addition, seven semi-structured interviews were conducted. The formulation of the questions was guided by the phases (planning, monitoring, controlling, evaluation) of SRL (Pintrich, 2000, 2004) and social interaction (i.e., social space, social presence, sociability) theory (Kreijns et al., 2020, 2021). Example questions are: “How did you experience the social presence of your peers during the monitoring phase?” and “In what way did you feel connected to your peers?”

### Procedure

The data were gathered in compliance with ethical norms; students gave active informed consent and participated voluntarily. The research was ethically approved by cETO the ethical committee of the Open Universiteit under number U202103810.

#### Questionnaire

In June 2021, all students in the Trends course were invited *via* the course website to participate in a scientific study about peer interaction. The questionnaire was made available *via* the open-source online survey tool Limesurvey.^1^ After 1 week and 2 weeks, a reminder was sent. Filling out the questionnaire took approximately 5 min.

#### Reflective Questions

For the peer feedback assignment in section three of the course, students needed to find their own peer feedback partner *via* the discussion board in the course. As students could choose which conference to visit, it was advised to find a partner who visited the same conference although this was not mandatory. For giving feedback, students assessed a review of a peer and provided feedback comments per criteria in the rubric. Students additionally described what changes they had made to their review based on the received peer feedback. After completing section three, students answered three reflective questions. Answering these reflective questions was conditional for completing the assignment and took approximately 5 min.

#### Interviews

All students who completed the Trends course since April 2020, were asked if they could be approached for an interview in the future and were asked for their email address. In June 2021, all students who indicated that they were willing to participate in an interview, were approached *via* email. After their indication of availability for an interview, 1-h appointments for an online interview *via* Microsoft Teams were scheduled. The interviews took approximately three quarters of an hour. After consent of the interviewee, the sessions were audio-recorded for the purpose of transcription. All seven interview recordings were transcribed for coding purposes.

### Analysis

The interview transcripts and reflective questions were analyzed by the thematic analysis method which focuses on identifying themes within the available qualitative data (Braun and Clarke, 2006; Maguire and Delahunt, 2017).

Within this method, we followed a theoretical thematic analysis approach at the semantic level. This means that our analysis was driven by a specific research question and that the themes were based on the “…explicit or surface meanings of the data…” (Braun and Clarke, 2006, p. 84).

**FIGURE 2:**
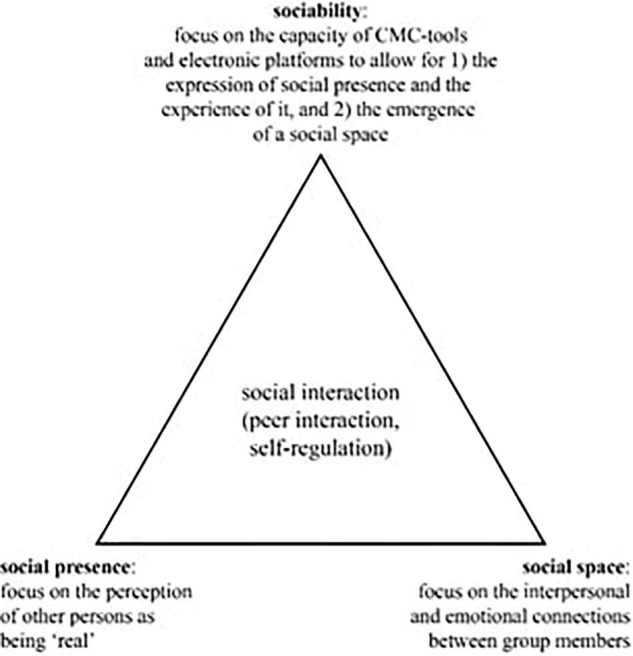
Final thematic map.

In stage one, the four researchers involved familiarized themselves with the data by reading and re-reading the transcripts and the reflective questions data. In stage two, the initial codes were determined by a pair of researchers on the topic of peer interaction in the four (planning, monitoring, controlling, evaluating) phases of SRL (Pintrich, 2000, 2004) and by another pair of researchers on the topic of the social interaction dimensions as present in the model of Kreijns et al. (2021). Each pair of researchers first coded a selected transcript separately after which the identified codes were discussed and modified. After consensus was reached, the transcripts were divided equally and coded individually with Dedoose.^2^ Subsequently, two researchers (one of each pair) identified, refined and defined internally homogeneous and externally heterogeneous themes emerging from the coded data which were relevant to the research question as well as relevant in the context of the entire data set. Further discussion and modification of the themes resulted in a final thematic map (see Figure 2). The thematic map shows the themes, the corresponding codes (between brackets) and the connection between themes.

In the last stage, all the thematic data was interpreted and analyzed within themes, between themes as well as holistically for the purpose of answering the research question.

For quantitative analysis, the social presence scale by Kreijns et al. (2020) was administered. The scale was developed using the Rasch measurement model (Rasch, 1960; Boone et al., 2014). Rasch analyses will produce Rasch person measures as “total scores” for the participants. These Rasch person measures can then be used as observed variables in any statistical analysis such as regression. Nevertheless, as the correlations between Rasch person measures and total scores for each dimension were found to be very high (respectively 0.998 for awareness of others and 0.977 for proximity of others) after performing the analyses, we decided to use total scores rather than Rasch person measures for easier interpretation. Subsequently, the data was used to calculate the mean scores per question. For the purpose of triangulation, the quantitative and the qualitative data were compared from a social presence perspective relating to the two dimensions of social presence (i.e., awareness of others and proximity of others; see section “Materials”).

## Results

To answer our general research question: “How are social space, social presence, and sociability supportive for peer interaction regulation in VLEs?,” we report the findings per phase of SRL following the four research questions. For each phase, students’ perceptions of social presence and social space as well as the sociability of the VLE are described in relation to the way they facilitate regulation of peer interaction. In this manner, the relationships between the concepts become evident. This method of working fitted our model (see Figure 1) and the exploratory nature of this research in which we were investigating student experiences. For each phase, we show in a holistic way how the concepts were related by using some illustrative quotes. After the description of the qualitative data per phase, the quantitative data reflect how students experienced social presence at course level. The leading constructs (social space, social presence, sociability, regulation of peer interaction) are shown in italics.

### Planning of Peer Interaction

In the planning phase, students’ awareness of *social presence* of peers was only marginal, resulting in a low degree of *regulation of peer interaction*. Also, they did not feel connected to their peers in this phase which indicates that the *social space* was vulnerable.

“But to say: I feel connected [to my peers], to go and work together with them? I didn’t have that at the time.”

For students the *sociability* of the VLE was not supportive for *social presence* of peers since the VLE functioned only as a first orientation on the course and a place for depositing materials:

“. You literally do not see anyone reacting to it [VLE] … So, then it [peers] remains a bit of a ‘flat figure’ so to speak.”

While in section three of the course, students had to do a compulsory peer feedback assignment (see section “Participants and Context”) which involved *regulation of peer interaction*, students already needed to take this into account in the planning phase. Here too, the *sociability* of the VLE did not work in a supportive way for *social presence* or *regulation of peer interaction*:

“At some point in your planning you have to make an appointment with someone to give you feedback [.]. I posted a message *via* [VLE], or even twice, and no one responded. Then I started looking back myself to see if anyone recently had sent a message.”

Because the *sociability* of the VLE was not perceived supportive for *regulation of peer interaction*, students looked for other forms of communication (mail, phone, social media) showing “real contact” (i.e., *social presence*), with WhatsApp being particularly mentioned by a majority of students because of its ability to react quickly:

“In [VLE], you always have to find an e-mail address or a telephone number to make real contact with someone [.]. Only when you have real contact *via* email or WhatsApp does it [*social presence*/*social space*] become better.”

Meeting each other (preferably face-to-face) contributed positively to *social presence* for students:

“Way before this conference, I was in touch with a fellow student *via* WhatsApp. We met in another course of this study. Beforehand, we shared which speakers we were visiting and it turned out that we were interested in the same speakers. During the writing process, my fellow student and I had a lot of contact *via* WhatsApp where we shared resources or talked to each other about the writing process.”

In summary, the planning phase appeared an important phase for the *regulation of peer interaction*. It is the start-up phase of the course and ideally requires *social presence* from fellow students to make *regulation of peer interaction* possible and develop a sound *social space*. Incorporating a task that requires cooperation with peers (e.g., peer feedback) contributed to *regulation of peer interaction* and the experienced *social presence*. A static VLE, intended only for informing students and posting materials, did not benefit for *social presence*, indicating that its *sociability* was low, and was therefore not supportive for *regulation of peer interaction* and for developing a sound *social space*. In order to make *regulation of peer interaction* possible, students themselves looked for additional communication channels that increased *social presence* as well as contributed to the *social space*. In this matter, they preferred to meet each other first (preferably face-to-face).

### Monitoring of Peer Interaction

In the monitoring phase, the students mainly used their own network for *regulation of peer interaction*. The need for contact (preferably face-to-face) within their existing network remained important in order to increase *social presence*:

“I think it was a very big advantage that I knew those fellow students beforehand. That I also saw them face-to-face during the conference.”

An increase in social presence also contributed to the *social space*:

“With some [I felt] more [connected] than with others.”

In addition to informal talks and consultation, discussions (*regulation of peer interaction*) also took place between these students:

“The contact you have is also because, what you have seen or experienced, you find interesting and you share that with each other. So of course, you talk about the content of such a conference. And that, of course, helps to focus or shape your own thoughts on the subject.”

This communication took place outside the VLE. The importance of the *sociability* of the VLE for *social presence* was therefore diminishing. When contact was more long-term (i.e., only when people have known each other longer), we also saw other forms of communication such as video calling. Overall, however, people preferred to communicate *via* WhatsApp because it is faster.

“Through WhatsApp it is ultimately much faster, then you can quickly ask a question that you have.”

Summarizing, in the monitoring phase we saw that the students were already on their way in the course and that their networks were already formed. The students knew each other for some time and especially meeting each other in “real life” (preferably face-to-face) contributed to *social presence*. In those networks, *social presence* further increased, which also had a positive effect on *social space*. The VLE was no longer needed to support *social presence* and *social space* since the students used other means of communication.

### Controlling of Peer Interaction

In this phase, the students had to provide peer feedback in pairs on their third assignment (see section “Materials and Methods”).

Most students chose to work with a peer they did not know from their network. This gave the VLE a floor again for making contact and agreements. However, as in the previous phases, communication subsequently took place primarily *via* email and WhatsApp and not *via* the VLE. The course was a self-paced course where students studied at their own time and pace without plenary moments, which sometimes made it difficult for students to find peers who were willing to give feedback and which made it more difficult to develop a sound *social space*:

“Because the start time of Trends is self-paced, I think the students feel less connected to each other. The willingness to give peer feedback is therefore much lower.”

As most students worked together with unfamiliar peers and as the *social space* was still vulnerable, some students experienced giving and receiving peer feedback in an online learning environment a bit stressful:

“In the beginning, I found it quite exciting to give feedback. I wanted to give the best possible feedback to the other person.”

Working with unfamiliar peers also had its advantages. The different and objective view of fellow students was particularly appreciated:

“I did find it positive to work with someone I didn’t know on this assignment. Was nice from a different perspective.”

Sometimes students regretted that the peer feedback did not lead to more in-depth work; as in previous phases, the hardly experienced *social presence* and low *sociability* of the VLE did not support or stimulate this:

“For me personally, it would be more interesting if there were more students on the forum with whom to discuss. I have hardly received any answers to the questions I asked my fellow students, which I understand since every student submits the assignment at a different time. However, I do think this is a pity.”

Some students gave peer feedback to fellow students from their own network; often they did so during earlier moments in the study:

“We worked in a group of three and gave feedback on each other’s work during the process. This kept us focused and above all motivated.”

In summary, the controlling phase findings show that giving and receiving peer feedback outside one’s own network supports *social presence* of “new” students and thus contributes to *regulation of peer interaction*. Since the students did not yet know each other, similar routines arose as in the first two regulation phases: (1) *social presence* appeared as a prerequisite for *social space* for the purpose of *regulation of peer interaction*; (2) due to its low *sociability*, the VLE did not support this process. Therefore, students were forced to use other means of communication for *regulation of peer interaction* purposes which increased *social presence* and *social space*. Because this was not a long-term process (students worked on this assignment only for a few months), *regulation of peer interaction* did not lead to in-depth conversations. When students within their own network provided each other with peer feedback, we saw that the processes from the monitoring phase deepened (more *social presence* leading to more *social space*, resulting into *regulation of peer interaction* toward deeper conversations). Despite the short-term nature of cooperation with “new peers,” this *regulation of peer interaction* was perceived by students as valuable, since the objective view of a peer improved the quality of their work.

### Evaluation of Peer Interaction

Some students became aware of the importance of *social presence* for the *regulation of peer interaction* for their personal development and therefore pursued it more actively:

“So, the whole idea behind social presence, that the presence of fellow students, whether it be digital, on the phone or actually physical, contributes to your [students’] learning, is something I experienced in [course] … I recognize the importance of it more strongly and therefore I look for it more actively.”

Visiting conferences (which contributed to *social presence*) had also proved valuable for students and was something they wanted to continue doing in the remainder of their study and after their study for the benefit of their network:

“Yes. For the content, but also to meet those people again, of course.”

Students also continued to use peer feedback because they have experienced that looking at work from different perspectives (*regulation of peer interaction*) contributes to their development.

“…. But if you receive feedback yourself, then you think: so! Then you know how it could have been done differently, so I keep that in mind now. I applied that immediately indeed.”

Overall, the results showed that students in the evaluation phase were aware of the importance of *social presence* and also indicated that they will pay attention to this in the continuation of their studies. The awareness of the importance of *social space* was not reflected in the findings.

### Social Presence Scale

**FIGURE 3:**
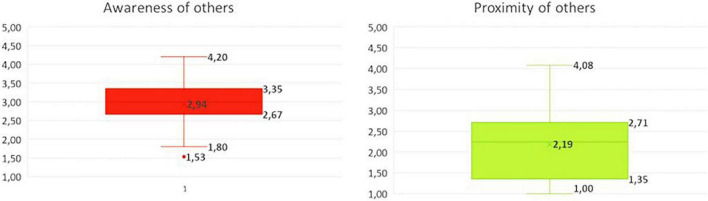
Distribution of mean scores for social presence sub dimensions “awareness of others” and “proximity of others” (*N* = 41). All items were scored on a 5-point Likert scale (1 = totally disagree, 5 = totally agree).

The results of the questionnaire (see Supplementary Table 1), which represent a course level view of the students on their experienced *social presence*, supported the qualitative findings related to their experienced *social presence*. Figure 3 shows that the majority of the students indicated that they were not particularly aware of others during the course (*M* = 2.94) and felt even less proximity of others (*M* = 2.19). The means related to “proximity of others” showed a fairly wide distribution, indicating that the views of the respondents were somewhat divided. Nevertheless, all means, but one outlier, indicated a negative feeling of the experienced proximity of others.

When looking at the mean scores per question (see Supplementary Table 1) it stands out that students felt that they were the only ones present in the VLE (“awareness of others” item 14) and that they did not have the idea that their fellow students were “real” to them (“awareness of others” item 13). Also, they did not feel at all as if they were in a face-to-face group (“proximity of others” item 5), that their fellow students were near (“proximity of others” item 2) or that their fellow students were around (“proximity of others” item 3).

As these questions only reflect the experienced *social presence* in the VLE, they do not account for any experienced *social presence* outside the VLE such as *via* WhatsApp or other communication channels as were used by the students alternatively due to the low *sociability* of the VLE.

## Conclusion

Regulation of peer interaction in online higher education deserves more attention as it is largely relied upon. Consequently, students who lack SRL strategies cannot learn successfully online (Eggers et al., 2021). According to Broadbent and Poon (2015), students’ abilities to self-regulate are also positively related to students’ academic achievement. Therefore, the focus of this explorative study was to gain insight into how peer interaction can be facilitated in the online setting of a master course in support of students’ self-regulation. While social space, social presence and sociability of the VLE are vital in order to stimulate students’ regulation of peer interaction, we explored the following overall research question: How are social space, social presence, and sociability supportive for peer interaction regulation in VLEs? If we have a better understanding of how students develop through the four phases of SRL, we may be able to better support peer interaction regulation in each of these phases.

Social presence of peers seems particularly important in the regulation phase of planning because this is the phase in which students make an action plan for their learning throughout the course where peer interaction plays a role. When the sociability of the VLE provides insufficient support in this process, students experience little social presence of peers which they compensate for by meeting each other (face-to-face and/or online) by using other communication channels such as WhatsApp that provided a semi-synchronic communication channel.

Whereas in the planning phase students needed more social presence in the VLE, in the monitoring phase we see that their impression of fellow students has now formed in their own networks. In those networks, the social presence is further increased by informal conversations, consultation and discussion and we also see an increase in social space. In line with these findings, earlier research (Sie et al., 2012) showed that social affordances of VLEs do not fit informal connections (social space).

In the controlling phase, the social presence and social space of peers in students’ networks further increases which accordingly supports the confidence to enable open discussions (long-term relationships). In some cases this is reinforced by some students meeting each other face-to-face at conferences. This may entail a risk that we see in networked learning: if the social space of the participants is highly sound, it can lead to the participants in the network moving too much along one another so that no innovative perspectives can emerge (Vermeulen, 2016). This is because strong learning relationships and common perspectives lead to more learning from improved routines (Vrieling-Teunter et al., 2021). On the other hand, peer interaction assignments that require short-term relationships with peers outside of students’ network (e.g., the peer feedback assignment in our course), provided opportunities to look at each other’s work more objectively, which resulted in quality improvement according to the students.

The analysis of the evaluation phase shows that students became aware of the importance of social presence for the regulation of peer interaction and that they actively look for it. The importance of social space is not specifically mentioned by the students, yet between the lines, students’ answers during the interviews showed that social space is important to deepen trust and to prevent tension for the benefit of peer interaction regulation.

## Discussion

Overall, this study shows that the sociability of the current setup of the VLE is inadequate for supporting social presence and subsequently social space and is therefore not supportive for the regulation of peer interaction within the online course. The fact that some of the students in this course met face-to-face at conferences they attended alleviated this lack of support of the VLE. However, often distance education contexts or courses do not support face-to-face contact. It is therefore important to investigate how we can adapt the sociability of VLEs to support this purpose and, accordingly, how the implementation of such interventions increases the experienced social presence and social space of fellow students and thus contributes to the regulation of peer interaction. To achieve this, the planning phase is particularly important because that is generally the moment to make contact with fellow students. If we expect of students to dialog with peers, for example in online discussion groups, the planning phase is key because this is the phase where goals are set and planning is done including collaborating with other learners in order to aid each other’s learning. We assume that the planning phase in other online higher education courses is also an important phase for guiding peer interaction regulation, but follow-up research could shed more light on this.

Although we did not specifically look for interventions to support social presence, social space and sociability to enhance regulation of peer interaction, during the interviews some students indicated that facilitation of peer interaction regulation in the VLE can be improved. Such interventions are important for instructors of online higher education to monitor and facilitate students in how the regulation of peer interaction is shaped. This can result in awareness of how peer interaction regulation is supported and where adjustments are needed to meet the intended course goals. One option mentioned was the use of pictures in the VLE to experience more social presence. However, the idea is controversial as Walther (1992, 1996) pointed out that the use of pictures only provides a limited impression of fellow students and may undermine long-term interaction. A second option mentioned for increasing social presence by students was to meet at certain times for a particular task to get acquainted. In this matter, most students prefer face-to-face contact, which is in line with the findings of Bruggeman et al. (2022). Their research shows the importance of a combination of online flexibility for the transfer of knowledge and face-to-face interaction for the benefit of social interaction. However, this is not always possible in online higher education. As for the facilitation of online regulation of peer interaction, students mentioned the possibilities of break-out rooms in online conferences for informal (e.g., getting to know each other better) and formal (e.g., discussing substantive themes with each other) purposes. Also, opening up a virtual classroom where students, with or without a supervisor, can discuss a substantive theme appropriate to the course is an interesting idea. Yet, in self-paced courses such as the one in which our research took place, this is difficult to organize because students work on the assignments at their own pace and time. But this is something we need to think about in order to accommodate students’ peer interaction in online higher education settings in support of their SRL. A technology-enabled approach for finding and recommending peers might be supportive for starting regulation of peer interaction (Van Rosmalen et al., 2008).

This study is an explorative investigation into the importance of social presence, social space and sociability for the regulation of peer interaction in online higher education. The exploratory nature has led to interesting findings, but there are also some limitations. First, we used a small sample limited to a self-paced course in higher online education. Future research should verify the findings with larger groups of students and in various contexts. Also, in this study we only measured social presence both qualitatively and quantitatively. In a subsequent study, it would be interesting to examine all variables in a mixed methods design. Third, we only interviewed the students afterward and asked them for their retrospective reflections. It would be interesting to monitor the regulation of peer interaction in the interim as well.

To end up, it is important for course designers to take the following principles into account when designing online higher education in order to support students in their regulation of peer interaction:

• Be aware of the degree of sociability of the VLE, especially in the planning phase; support the expression of social presence and the creation of a sound social space with peers; offer possibilities in the VLE and let students additionally use their own means of communication;
• Introduce opportunities for peer feedback to stimulate regulation of peer interaction as well as improvement of learning outcomes;
• Make students aware of the importance of social presence and social space in order to prevent tension and increase trust when regulating peer interaction.

## Data Availability Statement

The raw data supporting the conclusions of this article will be made available by the authors, without undue reservation.

## Ethics Statement

The studies involving human participants were reviewed and approved by the cETO Committee of the Open Universiteit under number U202103810. The patients/participants provided their written informed consent to participate in this study. Written informed consent was obtained from the individual(s) for the publication of any potentially identifiable images or data included in this article.

## Author Contributions

All authors have contributed and agree to be accountable for the content of the work.

## Conflict of Interest

The authors declare that the research was conducted in the absence of any commercial or financial relationships that could be construed as a potential conflict of interest.

## Publisher’s Note

All claims expressed in this article are solely those of the authors and do not necessarily represent those of their affiliated organizations, or those of the publisher, the editors and the reviewers. Any product that may be evaluated in this article, or claim that may be made by its manufacturer, is not guaranteed or endorsed by the publisher.

## Footnotes

1
http://www.limesurvey.org

2
http://www.dedoose.com

## Supplementary Material

Supplementary Table 1 Mean scores per question corresponding to respectively sub dimension “awareness of others” and “proximity of others” (*N* = 41). All items were scored on a 5-point Likert scale (1 = totally disagree, 5 = totally agree).
