# Supplementary material for: Facilitating Peer Interaction Regulation in Online Settings: The Role of Social Presence, Social Space and Sociability
Source: Front Psychol. 2022 Apr 25;13:793798. doi: 10.3389/fpsyg.2022.793798 (PMC9081834; doi:10.3389/fpsyg.2022.793798)
Supplement: Supplementary Table 1 — Mean scores per question corresponding to respectively sub dimension “awareness of others” and “proximity of others” (N = 41). All items were scored on a 5-point Likert scale (1 = totally disagree, 5 = totally agree). [file Table_1.DOCX]

|  | **Awareness of others** (Cronbach’s α = .92 ) | Mean |
| --- | --- | --- |
|  | Preambule: In this learning environment… |  |
|  |  |  |
| 1* | I only can get a glimpse of my fellow students | 2,46 |
| 2 | I can form distinct impressions of some of my fellow students | 2,83 |
| 3* | I know my fellow students are here too but I do not ‘see’ them | 2,49 |
| 4 | My fellow students are not abstract at all, which was what I first expected | 2,95 |
| 5* | I feel my fellow students are far away | 2,71 |
| 6* | I do not know who my fellow students are | 2,73 |
| 7 | It feels like as if I deal with ‘real’ persons and not with abstract anonymous persons | 3,46 |
| 8* | Nothing more than that I am aware of my fellow students | 2,59 |
| 9 | It feels as if all my fellow students are ‘real’ physical persons | 3,12 |
| 10* | Nothing more than that I feel distant from my fellow students | 2,61 |
| 11* | It feels like none of my fellow students are here | 3,29 |
| 12 | I am aware of my fellow students | 3,37 |
| 13* | My fellow students do not really live for me | 3,63 |
| 14* | I am the only one present | 3,71 |
| 15 | I feel none of my fellow students wants to communicate with me | 2,20 |
|  | **Reverse coded* |  |
|  |  |  |
|  | **Proximity with others** (Cronbach’s α = .94) | Mean |
|  | Preambule: In this learning environment… |  |
|  |  |  |
| 1 | I feel that I can see my fellow students right in the eyes | 2,05 |
| 2 | My fellow students are very near to me | 2,08 |
| 3 | I constantly feel that my fellow students are around | 2,08 |
| 4 | It feels as if all my fellow students and I are in the same room | 2,08 |
| 5 | It feels like if we are a face to face group | 1,80 |
| 6 | It feels as if all my fellow students and I are in close proximity | 2,03 |
| 7 | I am sure my fellow students are here too | 2,68 |
| 8 | I can really see my fellow students as if they were in front of me | 2,10 |
| 9 | I can make a clear picture of all of my fellow students | 2,10 |
| 10 | I feel a sense of my fellow students’ presence | 2,70 |
| 11 | I strongly feel the presence of my fellow students | 2,15 |
| 12 | All of my fellow students feel that I am a ‘real’ physical person | 2,45 |
